# Alignment of Consumers’ Expected Brain Benefits from Food and Supplements with Measurable Cognitive Performance Tests

**DOI:** 10.3390/nu16121950

**Published:** 2024-06-19

**Authors:** Hayley A. Young, Alecia L. Cousins, Carol Byrd-Bredbenner, David Benton, Richard C. Gershon, Alyssa Ghirardelli, Marie E. Latulippe, Andrew Scholey, Laura Wagstaff

**Affiliations:** 1 Department of Psychology, Swansea University, Wales SA2 8PP, UK; a.l.cousins@swansea.ac.uk (A.L.C.); d.benton@swansea.ac.uk (D.B.); 2 Department of Nutritional Sciences, Rutgers University, New Brunswick, NJ 08854, USA; bredbenner@sebs.rutgers.edu; 3 Feinberg School of Medicine, Northwestern University, Chicago, IL 60208, USA; gershon@northwestern.edu; 4 NORC, University of Chicago, Sacramento, CA 95811, USA; ghirardelli-alyssa@norc.org; 5 Institute for the Advancement of Food and Nutrition Sciences, Washington, DC 20005, USA; mlatulippe@iafns.org; 6 Nutrition Dietetics and Food, School of Clinical Sciences, Monash University, Notting Hill, VIC 3168, Australia; andrew@scholeylab.com; 7 Centre for Mental Health and Brain Sciences, Swinburne University, Melbourne, VIC 3122, Australia; 8 NORC, University of Chicago, Chicago, IL 60603, USA; wagstaff-laura@norc.org

**Keywords:** consumer terminology, mood, cognitive health, brain health, nutrition, diet, supplements, validated tests

## Abstract

Consumers often cite cognitive improvements as reasons for making dietary changes or using dietary supplements, a motivation that if leveraged could greatly enhance public health. However, rarely is it considered whether standardized cognitive tests that are used in nutrition research are aligned to outcomes of interest to the consumer. This knowledge gap presents a challenge to the scientific substantiation of nutrition-based cognitive health benefits. Here we combined focus group transcript review using reflexive thematic analysis and a multidisciplinary expert panel exercise to evaluate the applicability of cognitive performance tools/tasks for substantiating the specific cognitive benefits articulated by consumers with the objectives to (1) understand how consumers comprehend the potential benefits of nutrition for brain health, and (2) determine the alignment between consumers desired brain benefits and validated tests and tools. We derived a ‘Consumer Taxonomy of Cognitive and Affective Health in Nutrition Research’ which describes the cognitive and affective structure from the consumers perspective. Experts agreed that validated tests exist for some consumer benefits including focused attention, sustained attention, episodic memory, energy levels, and anxiety. Prospective memory, flow, and presence represented novel benefits that require the development and validation of new tests and tools. Closing the gap between science and consumers and fostering co-creative approaches to nutrition research are critical to the development of products and dietary recommendations that support realizable cognitive benefits that benefit public health.

## 1. Introduction

The consumption of foods, beverages, and supplements can influence brain functioning, which in turn may benefit cognition and mood [1,2,3,4]. Whilst there is a notable absence of peer reviewed literature on the topic, consumer surveys indicated that cognitive and affective outcomes are key motivations for dietary choices [5]. This consumer motivation could be leveraged to significantly benefit public health [6]. For example, evidence indicates that public health messages that align to the interest of the consumer are more likely to be taken up and acted upon [5,6,7,8,9]. Indeed, poor cognitive and mental health carries a significant burden on individuals, families, healthcare systems, and society as a whole [7]. Therefore, it is essential that consumers can comprehend and assess the functional properties of foods and supplements, and their role in optimizing or maintaining brain and cognitive health [8,9]. However, the terms that consumers use to articulate beneficial outcomes may have a variety of individual interpretations, which may or may not be easily substantiated by existing validated research techniques. For example, members of the public generally use terms such as ‘increased sharpness’ or ‘reduced stress’ to describe their motivations for changing their diets or taking supplements [5]. However, little is known about how consumers conceptualize these statements, or what they mean to the consumer regarding expected outcomes. It is also unclear whether scientific evidence for these expected outcomes exists, or even whether these outcomes can be verified by nutrition scientists. This knowledge gap presents a challenge to the scientific substantiation of cognitive health benefits and makes it difficult to communicate evidence-based recommendations to the public.

Consumer surveys and purchasing behaviour show a growing interest in dietary patterns, functional foods and beverages, and dietary supplements for maintaining or improving brain health. The global brain health supplements market was valued at USD 8.63 billion in 2022 and is expected to grow at a compound annual growth rate of 13.3% from 2023 to 2030 [10]. Almost a quarter of US adults aged 18 and above and more than one-third of US adults aged 74 and older consume at least one supplement to improve brain health, and 71% of those taking supplements aim to maintain or improve memory, and 60% aim to maintain or improve mental sharpness [11]. Mental health is another motivation with 16% of supplement consumers targeting mental health and 14% managing stress [12]. The 2023 US Food and Health Survey indicated that the top-sought benefits were increased energy (40% of users), improved sleep (27%), improved brain functioning (including memory, focus and cognition) (25%), and emotional/mental health (24%). Thus, consumers are attracted to foods and dietary supplements for their potential impact on cognition and mental health. In this context, it is crucial to understand how consumers view brain health and the functional role of nutrition in maintaining it.

Scientific substantiation of brain health benefits is crucial for promoting beneficial foods, beverages, and dietary supplements. Under Federal Trade Commission FTC regulations, companies must use reliable evidence from controlled human clinical trials. Validated cognitive performance tests evaluate aspects of memory, language, visuospatial abilities, executive function, and attention [13]. However, the alignment of these tests with consumer outcomes is not well documented. There is a need to accurately understand what consumers hope to achieve from functional foods, beverages, and supplements, especially regarding day-to-day benefits, so that it can be paired with clear information that is scientifically substantiated.

Research on consumer brain health literacy is limited. Surveys showed a lack of knowledge about dementia and mental disorders like schizophrenia, bipolar disorders, and autism [9]. Qualitative research indicated that some consumers define brain health as the absence of dementia or mental health challenges [14], while others describe the brain’s role in psychological processes or cognition [15]. Consumers use various lay expressions to describe brain health-related concepts, such as mental clarity, focus, or sharpness [5]. These terms are important as they reflect how consumers perceive and communicate their understanding of brain health-related concepts in a way that is understandable and relatable to them. However, whether validated tools align with consumer understanding is currently unknown.

Therefore, the aim of the present research was to: (a) better understand how consumers comprehend the impacts of nutrition on brain health; (b) determine the consumer terminology commonly used to describe these benefits; and (c) establish areas of alignment and misalignment between these descriptions and validated cognitive performance research tools. This research was aimed at contributing to the progress of the nutrition and cognition field by establishing approaches to create evidence-based messages that can benefit consumers who seek to enhance their cognitive health.

## 2. Materials and Methods

This research had two components: (1) qualitative consumer research that determined the benefits consumers expected from nutritional products, how they articulate these benefits in their own words, and how they expect these benefits to play out in everyday life, and (2) consideration by a multidisciplinary expert panel as to whether current cognitive performance tools/tasks can substantiate specific cognitive benefits articulated by consumers. The expert panel comprised scientists who specialise in cognitive health and the development of psychometrically robust measurements (RCG), the effect of diet and nutrition on cognition and brain health (HY, DB, AS), and translational consumer research (CBB).

**Part 1—Qualitative consumer research**

To provide an unbiased view of consumers knowledge and expectations about nutrition and brain health, this part of the research was designed and carried out by the independent research group National Opinion Research Center (NORC) at the University of Chicago under the supervision of AG, MPH, RD. Four focus group discussions were conducted with ‘mainstream’ consumers; that is, those who currently consumed foods, beverages, or dietary supplements to improve/support their brain health. Details regarding the types of foods/supplements participants were taking were collected as part of the screener and can be found at https://osf.io/xvhqj/ (URL accessed on 30 May 2024). The groups were split by age and gender as described below.

**Participants**

Participants were recruited through targeted social media advertising (Facebook Ads Manager) and the Oracle Data Cloud. Ads directed potential participants to a Qualtrics (Provo, UT, USA) survey (completed by 1176 individuals) to determine eligibility including demographics and health-related behaviors e.g., diet and supplement use (available at https://osf.io/xvhqj/). Inclusion criteria included being aged 19–59 y/o, using foods and nutritional supplements for brain health. Exclusion criteria for eligibility were designed to ensure unbiased attitudes, beliefs, knowledge, and behaviors toward healthy food and nutritional supplement usage (past or current work in the supplement or health food retail industry, as a nutritionist or dietitian, or having majored in nutrition). Additional information collected during the screening process to balance the sample included race-ethnicity, geographic distribution, and education level (available at https://osf.io/xvhqj/).

Forty-six individuals (22 aged 19–30 y/o and 24 aged 31–59 y/o; age ranges were based on the USDA Dietary Guidelines for Americans life stages) were recruited (see CONSORT chart in Supplementary Information: Figure S1). In the event, thirty-nine total respondents participated in four focus groups (19 aged 19–30 y/o and 20 aged 31–59 y/o). There were 12 males, 26 females, and 1 non-binary participant.

**Focus Group Moderators guide and procedure**

Led by AG, NORC developed a structured moderator’s discussion guide with input from IAFNS (available at https://osf.io/xvhqj/). The first part of the focus group gained an initial insight into how consumers describe ‘brain health’ in their own words, with minimal prompts from the moderator. They were then asked to talk about the effects of food or nutrition on brain health, and the kind of brain functions they are currently aiming to improve. Towards the end of the session, the moderator probed participants about key terms related to brain health that had not already been discussed by the participants. Each respondent was called on to provide input to all questions to ensure all were engaged in the focus group. The research protocol and moderator’s guide were reviewed by the NORC Institutional Review Board and received an exempt designation (22-09-972). All focus groups were moderated by the same researcher to ensure consistency. Focus groups took place virtually via the Zoom online conference platform to allow for participation from a diverse sample across geographic locations in September 2022. Each group session lasted up to 90 min. Each session was audio-recorded with participants’ consent, with files later sent out for professional transcription. Focus group participants received an incentive of $125 USD.

**Qualitative data analysis**

Focus group transcripts were analyzed using reflexive thematic analysis to identify themes relating to the way consumers comprehend the cognitive benefits of food, nutrition, and supplements, including terminology used to refer to brain health that could map to validated cognitive performance tools or tests [16,17]. In the first phase of analysis, termed familiarization, HY read and re-read the transcripts, making initial notes of observations, thoughts, and questions, that were then discussed with AC. The main observation at this initial stage of review was that consumers lacked the scientific language used by field experts to accurately convey cognitive health benefits, and instead spoke in day-to-day terms about what brain health meant to them and how it might benefit from diet/nutrition supplements. This influenced the approach used in the next stage of analysis—coding.

The approach to coding followed a mix of inductive and deductive orientations, with models like the Cattell–Horn–Carroll (CHC) model of intelligence (a factor analysis–based model, which describes the major (broad ability) and minor (narrow ability) sources or factors of individual differences captured by cognitive tests and was previously suggested to apply to nutrition research [18,19]) providing a partial lens through which we interpreted and made sense of the data. However, the analyses were not limited by these models allowing for additional ‘data-driven’ observations and themes. Most codes went beyond the semantic (surface-level) level to capture consumers latent (underlying) meaning. This was necessary to facilitate Part 2—a clear mapping to validated cognitive tests. However, as one of the goals was to understand how consumers comprehend brain health ‘in their own words’ we also kept a record of the layperson terms consumers used to describe each construct (Table 1). Microsoft^®^ Word for Microsoft 365 MSO (Version 2404 Build 16.0.17531.20152) was used for the coding, with each data (section of transcript that contained information relevant to the research questions) item entered a table with a column for codes. HY and AC coded each focus group twice, each in a different order to ensure all focus group transcripts got a similar depth and insight and that there was no residual carry over effects of familiarity from the first coding session to the second.

**Figure 1:**
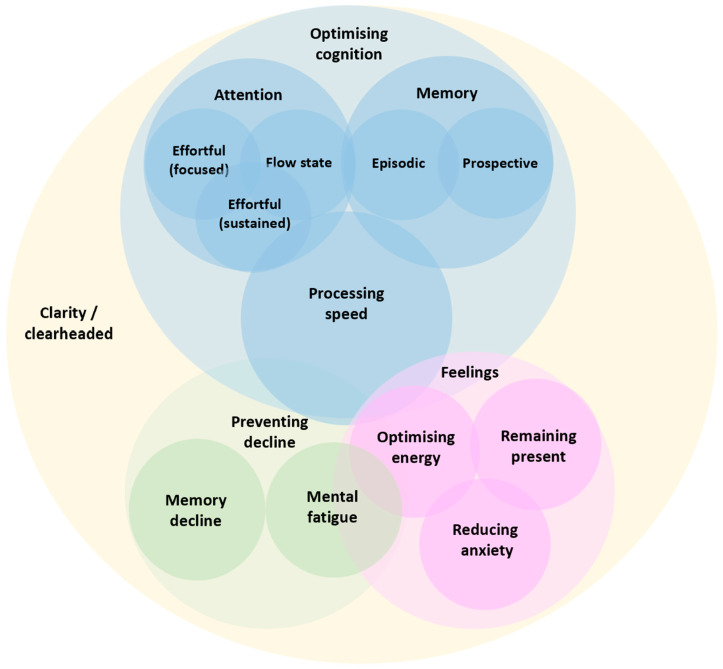
Consumer Taxonomy of Cognitive and Affective Health in Nutrition Research. Note that consumers often spoke about themes simultaneously. The hierarchical and overlapping structure is illustrated and represents consumers’ underlying associations and mental organization of how psychological processes are organized. This schematic allows for a linkage of consumer benefits to tools and tasks used to substantiate those benefits.

Theme generation was led by HY in discussion with AC. Microsoft PowerPoint (Microsoft^®^ PowerPoint^®^ for Microsoft 365 MSO (Version 2404 Build 16.0.17531.20152) was used to organise codes visually and develop a schematic representing how consumers mentally organised the psychological processes they regarded as being outcomes related to nutrition. The goal was to develop a schematic that allowed for a linkage of consumer benefits to tools and tasks used to substantiate those benefits. During construction of the schematic, based on familiarisation with the dataset, the hierarchical and overlapping structure of consumer responses was considered. This process led to three main themes based around the optimisation of cognitive functioning, preventing a decline in functioning, and feelings, moods, and mental health. Each theme also had two or three very clear and non-redundant sub-themes. An observation was that mental clarity was an important consumer concept that overlapped with (i.e., was discussed alongside) all other themes (Figure 1).

With the generation of this schematic, we moved to the final stages of reviewing identified themes and defining/naming themes. During refinement we again considered the overlap between themes and subthemes and potential redundancy. Our final conceptual themes to organize consumer perceptions of psychological processes they aim to influence using nutrition behaviors are illustrated in the Venn diagram shown in Figure 1. The schematic formed the basis of discussion by the expert group to link consumer benefits to tools and tasks used to substantiate those benefits (Table 1).

**Part 2—Alignment with validated cognitive performance research tools**

An expert group comprising AS, DB, RG, CBB and HY convened to determine the degree of alignment between consumer benefits and validated cognitive performance research tools. The process was led by HY who produced an initial mapping between consumer benefits and validated tests and tools. This was then circulated to the expert group for refinement and consensus. There were only minor disagreements which were resolved through discussion. Box 1, Box 2, Box 3, Box 4, Box 5, Box 6, Box 7, Box 8 and Box 9 and Table 1 presents the outcome of these discussions as well as terminology commonly used by consumers in each domain. A nuanced description of all available tests is beyond the scope of this manuscript and readers are referred to other authoritative documents on this topic [13,41,46].

## 3. Results

Following the Thematic Analysis of focus group transcripts, several key themes and subthemes were inferred from the data as listed in Table 1. To maintain anonymity, participants have been assigned pseudonyms.

### 3.1. Theme One: Optimizing Cognition

Consumers spoke of optimizing cognitive function, including three key subthemes: Memory, Attention and Processing (speed).

#### 3.1.1. Memory

Consumers often discussed “memory” when thinking about how they would describe brain health,

“*Well, brain health is something that improves memory, focus and clarity*”

P1, group 2

Memory was also a common facet of cognitive function that consumers aimed to enhance or optimize when taking supplements.

“*I’ll take omega threes, I’ll take various other supplements, but what I’m constantly thinking about is my memory*”

P2, group 1

Consumers discussed both episodic memory (the ability to recall past events) and prospective memory (the memory of planned events that will happen). Consumers noted that brain health means being able to recall events or retrieve information (clear examples of episodic memory):

“*Basically, being able to remember long time thoughts of if somebody had told me something about a year ago or maybe I learned something in school, I want to keep that forever in my memory because it might be something valuable*.”

P3, group 4

“*I am always and forever looking for my keys before we go somewhere. It’s like this, like, my brain health. I can’t remember ever where my keys are…*”

P4, group 4

Some consumers discussed memory in terms of their ability to remember tasks that need to be completed as part of their day-to-day routines (prospective memory):

“*Memory is just remembering all the things that I have to do in a day not missing doctor’s appointments, not missing my work zoom calls, making sure I am where I’m supposed to be…*”

P5, group 4

Box 1 **Expert assessment: Memory.**

Consumer terminology and everyday examples aligned well with those used by experts. Episodic memory can be reliably assessed using a standard word list test such as the Californian verbal learning test (CVLT) [20], or story recall such as the East Boston memory Test [21] which are frequently used in nutrition research [47] However, prospective memory has rarely been studied in relation to diet/nutrition therefore it is currently unclear whether the limited available tests (e.g., Cambridge Prospective Memory Test [25]) have the appropriate degree of sensitivity, and most have only been validated in clinical populations [48].

#### 3.1.2. Attention

Focused (or selective) attention was discussed often. Consumers were often aiming to improve their ability to focus selectively on one task or stimuli and in the presence of other distractions to complete daily tasks. In several examples, participants described this process as effortful.

“*…I need to be focused because I need to be as productive as I can because coding is painfully slow even in the best of situations, …I almost feel like there’s a clipboard on a computer when you clip something, I’d like my brain to be able to hold a thousand clips because I’m having to prioritize and deprioritize different chunks of information all day long…*”

P6, group 2

“*I can easily just get distracted. So for me, brain performance, being able to stay focused and pay attention to what I’m doing, and not easily check my phone and get distracted with that*.”

P7, group 3

However, in contrast, focused attention was also referred to as being “in the zone” which reflects a state of flow, where focused attention is not considered effortful:

“*I’m mainly trying to improve my focus and my memory… For me, focusing is focusing on one task at a time completing it and then moving on to the next thing. I’m being completely immersed in what I’m doing at the moment and staying on task*.”

P5, group 4

Some consumers also discussed their intention to improve their ability to engage in sustained attention, where attention is focused and deployed for a prolonged period of time.

“*…Like time management…focusing in one task for more than 15, 20 min. Being able to start and follow through on the task instead of starting so many of them and then not really finishing them…*”

P8, group 4

Box 2 **Expert assessment: Attention.**

Focused attention is the ability to respond discretely to specific visual, auditory, or tactile stimuli. Selective attention is similar and is the ability to selectively attend and then respond to specific, important stimuli whilst ignoring other irrelevant stimuli. Although the two forms of attention are subtly different, consumers used the term ‘focus’ to describe both kinds of attention, however, this does align with expert use of the terms as they are often used interchangeably in the scientific literature. Both require effortful attentional control to filter out distractions. Well validated tests include the Eriksen Flanker test [24], the Stroop test [23], and the Focused Attention Task [49] are available and commonly used in nutrition research.

Sustained attention is the ability to maintain a consistent behavioural response during continuous and repetitive activity. Again, consumers tended to refer to this kind of attention as ‘focus’ even though their descriptions fit the definition of sustained attention. This domain is often examined in nutrition research using tests such as the Continuous Attention Test [26] Sustained Attention Task [27].

When consumers spoke about being “in the zone” this described a concept in psychology known as a “flow state”; a mental state in which a person is completely focused on a single task or activity. It is characterised by a lack of time awareness and an unawareness of the self. This kind of focused attention feels effortless [50]. Hitherto, no study has considered the effect of diet/nutrition on the propensity to enter flow states. Therefore, it is unclear whether existing tests have enough sensitivity to detect effects of diet/nutrition. However, numerous well validated scales do exist such as the Flow State Scale (designed to assess the experience of flow during a particular activity) and Dispositional Flow Scale (designed to assess the typical frequency of flow experience during participation in an activity) [25].

#### 3.1.3. Processing Speed

A further subtheme was processing speed, where participants discussed the importance of being able to process information or stimuli quickly:

“*I use a lot of numbers and spreadsheets, so a lot of the times I feel like I could get confused or just feel a little bit slower throughout the day if I don’t take the supplements that I take*”

P9, group 1

“*But there’s also like that day to day like avoiding brain fog and being able to think quickly*.”

P10 group 4

“*I think like I mentioned before, is being able to think quickly because if you’re in a meeting, a live thing, and you take too long to think about things, then the conversation has gone on and you took too long to think about it. That’s why I want to think faster and be more nimble*.”

P4, group 4

Consumers also used the term “sharpness” which appeared to reflect a desire to process information more quickly:

“*Sometimes I think it’s hope in a bottle. I’ll take omega threes, I’ll take various other supplements, but what I’m constantly thinking about is my memory, my sharpness, my ability to obtain information and to be able to access it as quickly as I used to*”

P2, group 4

Box 3 **Expert assessment: Processing speed.**

Processing speed is the length of time it takes to perceive information, process information, and formulate or enact a response and is frequently assessed in diet/nutrition research. It is most often operationalized using reaction time measures on a range of tasks such as simple and choice decision times [47]. With other tests, speed of responding may depend on the nature of the of stimuli being processed, such as the Paced Auditory Serial Addition Test (PASAT) (auditory arithmetic ability) [29] or the Trail Making Test Part A (visual search/attention/motor speed) [30]. As such it cannot be assumed that a common unitary process underlies performance on these tasks. In addition, many cognitive tests that assess speed of processing (reaction time) also measure processing accuracy. Therefore, an important consideration is the ‘speed-accuracy’ trade off. That is, if participants are instructed to try to perform faster, they will become less accurate. Conversely, if they are instructed to perform more accurately, they will become slower. In the absence of any instruction participants will vary in what they prioritize depending on their individual predispositions. Consumers tended to use the term quite generally and lacked a nuanced understanding of these processes.

### 3.2. Theme Two: Feelings

When exploring participants’ reasons for taking supplements, consumers described taking supplements to manage feelings of anxiety (sub-theme one), to optimize levels of subjective energy (sub-theme two) and to remain present and aware (sub-theme three).

#### 3.2.1. Reducing Anxiety

“*I just wanna take more brain supplements and just eat better foods just for the brain as more of an anxiety thing… I’m always trying to find new ways to calm my anxiety down*.”

P11, group 1

Consumers related anxiety to a reduced ability to focus attention indicating that to consumers these two concepts were related, and that consuming supplements to reduce anxiety may have positive consequences for further aspects of cognition.

“*For me, it’s more so just anxiety and that’s why I mentioned the mental health piece because I feel like that affects my ability to be able to focus and do what I need to do because I am either worried about something that I am saying or decision that I am making*,”

P12, group 4

Coping with daily stressors also arose frequently in this theme:

“*I have tried Ashwagandha and it didn’t have any effect on me at all. I did hear that it was good for managing stress and I don’t like taking pills and drugs or anything. I don’t like being on anything, so I do look for supplements to help with whatever I can and it didn’t do anything for me, so I stopped taking it*”

P22, group 1

Box 4 **Expert assessment: Anxiety.**

Nutrition researchers often measure subjective anxiety using a range of self-report tools such as the State Trait Anxiety Inventory [31]. However, it should be noted that measures which were originally developed to measure clinical differences may not be sensitive to the relatively small effects of diet. A tool that may be able to detect more subtle changes in anxiety include the Profile of Mood States (POMS) Anxiety subscale [33]. The Positive and Negative Affect Schedule (PANAS) [34] has also been used in nutrition research, however, the scale contains items which may be less sensitive to diet e.g., “ashamed”, and sums to a “negative affect” score rather than anxiety per se.

An interesting observation was that consumers spoke of wanting to regulate stress and anxiety. In the psychological literature stress is most often defined as a mismatch between the demands of a task/context/stimuli and perceived ability to cope. The Perceived Stress Scale [35] has been used to capture this kind of emotional reactivity, and refers to one’s general responses over the preceding month. Subjective and physiological stress reactivity (cortisol, heart rate, skin conductance) to a standard laboratory stressor e.g., Trier Social Stress Test [36] or mental arithmetic [51] have also shown sensitivity to nutritional interventions. In addition, transient states may be more realistically captured using ecological momentary assessment (EMA) or experience sampling methods (ESM) [52].

#### 3.2.2. Optimizing Energy

Consumers commonly noted that increasing energy levels was a reason for taking supplements. Participants also noted experiencing increased levels of energy after taking supplements. With specific reference to vitamin B12, P12 [group 4] noted:

“*It helps you to get energy, helps with your mood but for me, I don’t drink coffee, so I usually take B12 and that’s a way for me to get energized, but also just feel charged up, which helps my mood*.”

P12, group 4

P12 indicates that taking this supplement enhances feelings of energy which has an impact on her mood, demonstrating that the consumer views energy as a facet linking to other areas of psychological wellbeing.

Box 5 **Expert assessment: Energy.**

Authors studying nutrition have differentiated three components of mental energy: a cognitive aspect (vigilance), motivation (to engage in cognitive work), and mood (feelings of energy) [44]. Consumers aligned on all three. Sustained attention and vigilance tasks are usually recommended for measuring the cognitive component [44,45], while the mood component tends to be assessed using the Vigor subscale of POMS [33] or Bond-Lader Visual Analogue Scales e.g., Lethargic-Energetic [44]. The motivational component is more difficult to assess as motivation needs to be inferred from other aspects of behaviour. This can be achieved from physical performance measures such as hand dynamometer or bicycle ergometer tasks, assuming a direct metabolic effect can be ruled out [41]. Motivation can also be assessed by measuring the amount of time that an individual is willing to persevere with a mental task e.g., during a free recall task [47]. Finally, some psychophysiological measures such as an increase in high frequency heart rate variability (0.15–0.40 Hz) might indicate task-related self-regulatory effort [42,53,54], and were influenced by diet [54].

#### 3.2.3. Remaining Present

Maintaining a sense of presence requires individuals to be fully aware of one’s own subjective experiences of their environment. Having a sense of being “present” or “in the moment” was a commonality in consumer descriptions of perceptions of brain health and reasons for taking supplements.

“*Yeah, when I think of brain health, I think of—also about like the mood, and also staying present. And like saying, just staying completely present in what’s going on right now*.”

P13, group 2

“*But being in the moment, being present and feeling, calm and ease about the next minute. You’re not even thinking about that, you just kind of living life, and just feel—like kind of at peace*.”

P13, group 2

“*For me, it’s about finding a supplement that will help me just be more in the moment in present because a lot of times I get very distracted by other things subconsciously. Sometimes, I don’t even realize it*.”

P12 group 4

Box 6 **Expert assessment: Presence.**

Psychologists have defined the concept of presence in a variety of ways, and this lack of consensus has extended to the way presence is operationalised. Generally, presence is the state of being fully engaged in the present moment, without being distracted by thoughts or feelings about the past or future. This definition aligns well with consumers use of the term. Several mindfulness questionnaires have subscales which could be used to measure presence. For example, the Five Factor Mindfulness Questionnaire Action Awareness subscale has items such as “I find it difficult to stay focused on what’s happening in the present” (reversed) [38]. However, whether such measures are sensitive to the effects of diet/nutrition are not known. In addition, researchers interested in these areas should consider reverse causality given that mindfulness has been associated with obesogenic eating styles [55]. A consideration is that contemporary models of presence emphasize mind-body awareness in that the feeling of presence is thought to arise from being able to accurately predict ones interoceptive signals [40]. As such recent findings of the effect of diet on interoception measures could be of interest [39].

### 3.3. Theme Three: Preventing Decline

A third theme was preventing a decline in cognitive function. This then was divided into two subthemes: preventing decline in memory (long term) and mental fatigue (decline over the short term).

#### 3.3.1. Preventing a Decline in Memory

Several participants referred to wanting to prevent decline in memory and protect their current level of function.

“*Yeah. I’m finding that since my teen years, my memory is not exactly what it used to be. I tend to be at a loss for words and try to remember people’s names when I never had that issue before so I’m always looking for something to improve my physical brain health*.”

P22, group 1

“*I don’t want to lose long-term and short-term memory that I do have now, so, that I can keep it going forward in my career. Even just with family stuff too, you know, remembering childhood memories that are precious to me or memories that I am making now with my family, I definitely don’t want to lose those in future*.”

P3, group 4

Preventing decline is also discussed in relation to dementia as well by some participants, such as P10 (group 4) who stated that when thinking of brain health, avoiding neurodegeneration was the main concern that came to mind:

“*So, the primary thing that comes to mind is long term brain health, like avoiding dementia…*”

P12 group 4

Overall, consumers tended to be concerned with preventing a decline in memory in the “long-term” to maintain function, retain long-term memories and reduce risk of neurodegenerative disease.

Box 7 **Expert assessment: Memory decline.**

Memory decline is challenging to assess as it requires that memory be assessed over a long period of time, often years or decades. Common tests that are used to measure general cognitive decline include the Mini-Mental State Exam (MMSE) or Montreal Cognitive Assessment (MoCA). Whilst such tests commonly appear in the nutrition literature, they may lack sensitivity to dietary-induced changes [46]. Some authors have also related diet to self-reported memory loss [56,57]. Whilst such measures sometimes predict actual declines in cognition [58,59], they might be more strongly related to depressive symptoms [59]. Finally, it was suggested that The National Adult Reading Test (NART) [60] or the Wechsler Test of Adult Reading (WTAR) [61], may provide an index of pre-morbid cognitive ability which can be compared with current functioning to provide a measure of decline [62]. This approach is based on the assumption that crystallised cognitive abilities (e.g., vocabulary, general knowledge) are preserved during aging fluid abilities (e.g., learning new information, problem solving) are not [63].

#### 3.3.2. Mental Fatigue

A second sub-theme related to decline was the desire to prevent short term decline that was associated with mental fatigue. Note that reducing fatigue was qualitatively different to optimizing energy (described above). That is, increasing energy reflected having above average level of energy or ‘a boost’, whereas reducing mental fatigue reflected maintaining a steady baseline. For example,

“*I definitely feel that afternoon slump where you just feel kind of tired, you have that brain fog, you can’t really concentrate so taking the supplements, watching what I eat, making sure I’m getting all the right vitamins and minerals, it really helps me to kind of fight this and stay focused…*”

P14, group 1

Consumers also spoke of mental fatigue in the context of “brain fog”. For example,

“*I don’t really get it. I get it more from when I am physically exhausted as to where I get like my brain gets tired from thinking. I don’t really get it then I just get a headache but I get a brain fog when I am exhausted like out of it to where I am just nothing firing, I am just drawing blanks*.”

P15, group 3

Box 8 **Expert assessment: Mental fatigue.**

Mental fatigue can occur during a prolonged test battery; therefore, the order of test presentation is important. Mental fatigue can be assessed by considering the decline in performance on long duration tests such as the continuous performance test, or during performance hampering conditions such as sleep deprivation, late in the evening, or during the post-lunch dip. However, a consideration is that this type of outcome may be moderated by participant effort and motivation, which complicates interpretation [41].

The subjective feeling of fatigue can be assessed using task difficulty or effort ratings such as the NASA Task Load Index [64], or the fatigue subscale of POMS [33] or Bond- Lader VAS (Alert-Drowsy, Mentally Slow-Quick Witted) measured before/after cognitive testing [65]. Like other subjective states, mental fatigue, tiredness, or sleepiness can also be assessed throughout the day using ecological momentary assessment (EMA) or experience sampling methods (ESM) [52].

### 3.4. Theme Four: Mental Clarity (Clear-Headedness)

Mental clarity or clear-headedness was a final overarching theme that consumers discussed in relation numerous other concepts including focus, and being mentally sharp:

“*I just really just think of it as just being—just having clarity. Just having a sharp mind, being able to remember things and staying on your toes*.”

P16 [group 1]

“*It’s basically a focus. You’re able to actually have a clear mind, clear thoughts, and be able to just express without having too much on your plate or being distracted*.”

P4 group 4

Box 9 **Expert assessment: Mental clarity.**

Mental clarity or clear-headedness is likely to comprise both cognitive (i.e., the ability to think clearly and focus) and affective (i.e., emotional clarity, the ability to identify what one is feeling) dimensions. However, hitherto no framework has yet been developed to guide measurement in this area. Here consumers were mostly aligned with cognitive clarity. In nutrition research mental clarity is often assessed using the clearheaded/confused subscale of POMS [33], or the Bond- Lader VAS (Muzzy-Clear headed). Whilst these measures have proven sensitive to nutritional interventions [66,67], this is an area that could do with further conceptual and methodological development.

### 3.5. Thematic Structure

Whilst the themes present here were distinct consumers often spoke about more than one concept simultaneously. Figure 1 illustrates the hierarchical and overlapping structure of the themes representing a “Consumer Taxonomy of Cognitive and Affective Health in Nutrition Research” (Figure 1). This schematic allows for a linkage of consumer benefits to tools and tasks used to substantiate those benefits.

## 4. Discussion

This study aimed to understand how consumers conceptualise the effects of diet on brain health and whether this aligns with current tests. Consumer responses centred on three themes: optimizing cognition, preventing decline, and feelings. Sub-themes included improving memory, attention, and processing speed, regulating anxiety, increasing energy, and feeling present. An overarching concept was mental clarity (Figure 1). This hierarchical structure was used to construct a Consumer Taxonomy of Cognitive and Affective Health in Nutrition Research. Experts agreed that for many of these consumer concepts well validated cognitive tests are available to demonstrate the effects of foods/nutrients (Table 1). However, gaps remain where there has been little attempt to validate tests and/or understand the effects of nutrition, for example, prospective memory, presence, and flow. This misalignment between consumer benefits and the scientific substantiation of those benefits is problematic and hinders evidence-based recommendations.

### 4.1. Consumer Terminology

The consumers showed a fair understanding of the aspects of brain health they hoped to improve/maintain using nutrition. However, various terms were used. For example, regarding the speed of information processing consumers often spoke of increasing ‘sharpness’ or reducing ‘brain fog’ or ‘slowness’. This use of ‘layman’ terms was common, for example many consumers spoke of being ‘in the zone’. Psychologists refer to this phenomenon (an extended period of effortless attention) as a flow state. Consumers often used similar yet distinct terms as though they were synonymous. For example, ‘focus’, ‘concentration’, and ‘attention span’. Sometimes the standardized vocabulary to describe certain concepts evaded consumers entirely; consumers instead explained by relating concepts to everyday activities. For example, needing to ‘prioritize and deprioritize different chunks of information’ in their job; a process psychologists call ‘selective attention’. From a phenomenological perspective it was clear that consumers experienced many aspects of brain health as overlapping and intertwined (Figure 1). For example, ‘mental clarity’ was mentioned as coinciding with numerous other terms including ‘focus’, ‘energy’, ‘sharpness’, ‘calmness’, and ‘memory’. Such fuzzy conceptual boundaries may not easily map onto existing empirically derived cognitive taxonomies. Therefore, we took a deductive approach to derive a “Consumer Taxonomy of Cognitive and Affective Health in Nutrition Research’ (Figure 1). This framework could be used to increase alignment between consumer benefits and the scientific substantiation of those benefits. As previous research has shown that consumers often do not understand complex scientific terms such as those used by European Food Safety Authority (EFSA) [68], this approach may also facilitate the communication of evidence-based recommendations to the public by helping communicators relate new materials to existing anchors within consumers cognitive structures [69].

### 4.2. Validated Tests

Experts agreed that for many of the domains discussed there are valid and reliable tools that can scientifically substantiate the effects of foods/supplements (Table 1). Areas of good alignment included ‘episodic memory’, ‘focused attention’, ‘sustained attention’, ‘subjective energy’ and ‘anxiety’. Well validated tests with proven sensitivity to nutritional interventions are available in these domains (see examples in Table 1). One caveat is that many lab-based psychological tests lack veridicality (the degree to which test scores correlate with measures of real-world functioning) and verisimilitude (the degree to which tasks performed during testing resemble those performed in daily life) which is clearly an important consideration when considering consumer benefits with relevance to everyday life [70]. The development of ecologically valid paradigms using advances in VR/augmented reality and/or ecological sampling may be able to overcome these limitations [71].

A further consideration is that the consumer taxonomy identified here (Figure 1) is less granular than those recommended by experts, for example the CHC model of intelligence [18]. This could be important as the cognitive architecture generally, and the psychometric properties of some tests, are still debated. For example, task-based measures designed to measure the same latent concepts such as the Stroop test [23] and the flanker task [24] (both tests of focused attentional—specifically attentional inhibition), often do not correlate, thus contradicting unified concepts of inhibition [72]. As there are various models of human cognition, each with different degrees of granularity [73], there is no objective reality with which to compare our consumer taxonomy and assess their level of understanding. Nonetheless, it is likely that consumers lack the detailed knowledge necessary to properly evaluate the cognitive health benefits of nutritional products. This has clear implications for both policy makers and the food industry concerned with communicating nutritional science to the consumer.

### 4.3. Untapped Consumer Benefits

Consumers described numerous cognitive subdomains which hitherto have not been investigated regarding nutrition. For example, when discussing memory, consumers were interested in remembering to do things in the future (prospective memory) as well as remembering the past (episodic memory). In nutritional research episodic memory (i.e., remembering things that have already happened) is one of the most frequently studied domains, and is usually operationalised using a Free word recall task e.g., Californian Verbal Learning Test [20], or story recall e.g., East Boston Memory Test [21]. Prospective memory is rarely assessed, possibly due to the absence of a suitably objective and standardised measurement tools. Nonetheless, given its obvious everyday significance e.g., remembering to take medication, remembering a medical appointment etc. this will be an important area for tool development and future nutrition research.

A similar gap concerned consumers description of presence and flow. In contrast to traditional lab-based tests of focused or sustained attention e.g., Stroop Test or Continuous Performance Test, flow is characterised by effortless attention. That is, the experience of being so absorbed by an engaging, enjoyable task that it completely holds one’s attention without the need to try and remain focused. Generally, there is also a loss of the sense of time and self-consciousness. Notably, flow states are most likely to occur when there is a match between one’s skill level and the difficulty of the task [25]. The propensity to experience flow states has been associated with a range of physical and mental health outcome making this an important area for future research [74]. Interestingly, given that some aspects of diet are reported to influence subjective task difficulty [75], it is theoretically plausible that diet may similarly influence the propensity to experience flow states. However, as flow is an experiential and multidimensional state, empirically testing this possibility may prove challenging. Whilst several self-report questionnaires and experience sampling procedures have been developed to operationalise flow [74], these are yet to be validated in regard to nutrition. Therefore, an important next step towards bridging the gap between scientific research and consumer expectations will be the development and validation of tests and tools for scientifically assessing these untapped consumer domains.

### 4.4. Strengths and Limitations

This study had several strengths. This research addressed a major challenge to the scientific substantiation of nutrition-based cognitive health benefits and identified areas in need of further conceptual and methodological development. A multidisciplinary expert panel was chosen to ensure consensus from the different perspectives. Both younger and older groups were included to ensure a complete picture of different age groups. A limitation was the small sample size. Whilst small samples are common in qualitative research it is possible that the present findings may not translate across cultures. Challenges during the focus groups included the ability to probe deeply enough on the day-to-day cognitive benefits. Consumers had difficulty explaining precisely what they expected and lacked the scientific terminology to describe behavioural phenomena. Consequently, a partially deductive thematic analysis was necessary and latent themes were inferred from the data [16]. Although deductive analyses can be biased towards theoretical preconceptions, the reflexive approach accepts that research is an inescapably subjective process that requires reflexivity. This requires acknowledging that HY is a trained psychologist with 10 years of postdoctoral experience of studying nutrition and cognitive health which will have shaped her engagement with the data, and the knowledge generated through this research. However, AC independently verified themes and subthemes and agreement regarding themes and subthemes was reached on discussion between AC and HY.

## 5. Conclusions

For the first time, we documented how consumers comprehend the potential benefits of nutrition on brain health. Taking a deductive approach, we derived a “Consumer Taxonomy of Cognitive and Affective Health in Nutrition Research” (Figure 1), which depicts the cognitive and affective space through the eyes of a consumer. An expert panel convened to determine the degree to which consumer benefits can be demonstrated by validated research tools. Areas of alignment (focused attention, sustained attention, episodic memory, energy levels, and anxiety) and misalignment (prospective memory, flow, and presence) were identified (Table 1). The present findings illustrate how consumer science can be leveraged to better understand the relevance of cognitive assessment tools to everyday life, support the scientific substantiation of consumer benefits, and enhance understanding of what can be delivered by foods, diets, and nutrients in terms of cognitive health. Future research aimed at developing tests and tools that better reflect consumer expectations, combined with targeted communication strategies to increase consumer knowledge and understanding, will be key to closing the gap between scientific research and consumer expectations.

## Figures and Tables

**Table 1:** Examples of validated tools for assessing cognitive and affective consumer benefits.

Theme	Sub-Theme 1	Sub-Theme 2	Sub-Theme 3	Consumer Terminology	Example Tests	Challenges/Future Directions
Optimising cognition	Memory	Episodic memory	-	“Memory” e.g., “remembering what has happened over the past few days”	Free word recall task e.g., Californian verbal learning test (CVLT) [20], or story recall such as the East Boston memory Test [21].	Validated for use in nutrition trials
Optimising cognition	Memory	Prospective memory	-	“Memory” e.g., “remembering what needs to be done”	Cambridge Prospective Memory Test [22]	Rarely tested regarding nutrition Test requires validation in nutrition
Optimising cognition	Attention	Focused/selective attention	Effortful attention	“Concentration” “Focus”	Stroop Task test [23], Arrow Flankers Task [24]	Validated for use in nutrition trials
Optimising cognition	Attention	Focused/selective attention	Effortless attention (flow)	“Being in the zone”	The Flow State Scale’ [25] Dispositional Flow Scale [25].	Flow states tend to occur in situations where skill matched by challenge (optimum difficulty level) Rarely tested regarding nutritionTests requires validation in nutrition context. New test may need to be developed
Optimising cognition	Attention	Sustained attention	-	“Concentration” “Focus”	Continuous Attention Test [26] Sustained Attention Task [27]	Validated for use in nutrition trials
Optimising cognition	Processing speed	-	-	“Sharpness”	Simple and choice reaction times [28] Paced Auditory Serial Addition Test (PASAT) [29] Trail Making Test Part A (visual search/attention/motor speed) [30].	Validated for use in nutrition trials Potential speed- accuracy trade-offs should be considered.
Feelings	Reducing anxiety/Maintaining a sense of calm	-	-	“Anxiety” “Calm” “Stress”	State Trait Anxiety Inventory [31]. The Hospital Anxiety and Depression Scale [32] POMS anxiety subscale [33] PANAS, (Watson [34]. Bond- Lader VAS (Tense-Relaxed) The Perceived Stress Scale [35]. Physiological reactivity (cortisol, heart rate, skin conductance) to stressor e.g., Trier Social Stress Test [36]. Ecological sampling of events and associated affective responses [37].	Consumers often spoke of maintaining calmness in the face of daily challenges. Nutritional scientists have tended to measure mood/anxiety either ‘in general’ or as part of a test battery. It may be more meaningful to assess emotional reactivity using a ‘stress test’ or ecologically sampling daily affect.
Feelings	Feeling present	-	-	“Feeling present” “In the moment” “In my body”	5-factor mindfulness questionnaire action awareness subscale [38] Heartbeat detection tasks to measure interoception [39]	Unclear whether mindfulness type measures will be sensitive to the effects of diet. The feeling of being ‘present’ can be assessed using novel VR technology although not been used in nutrition research to date. ‘Interoception’ or mind-body connection is thought to underlie ‘presence’ [40] but this is an emerging area of research.
Feelings	Mental energy (cognitive, motivational, mood)	-	-	“Energy”	Mood—Vigor subscale of POMS [33]. Bond-Lader VAS (Lethargic-Energetic). Time willing to persist on challenging task [41] HF-HRV effort [42,43].	Authors studying nutrition have tended to differentiate three components of mental energy: a cognitive aspect (vigilance), motivation (to engage in cognitive work), and mood (feelings of energy) [44]. Furthermore, mental energy is distinct from the physical energy needed to complete a task [45]. However, consumers did not make this distinction and tended to use the term quite generally.
Preventing decline	Short-term decline	Mental fatigue	-	“Tiredness” “Brain-fog”	Fatigue subscale of POMS [33] Bond- Lader VAS (Alert-Drowsy, Mentally Slow-Quick Witted) VAS ‘tired’ before/after cognitive tests to assess fatiguability	Whereas ‘energy’ was about general optimisation (an increase above baseline), ‘tiredness’ was about avoiding a decline below baseline e.g., after a long day
Preventing decline	Long-term decline	Memory decline	-	“Memory loss”	Free word recall task e.g., Californian verbal learning test (CVLT) [20], or story recall such as the East Boston memory Test [21]. examined over time to determine decline	Mini-Mental State Exam (MMSE) or Montreal Cognitive Assessment (MoCA) may lack sensitivity to dietary-induced changes [46].
Other	Mental clarity/clear-headedness	-	-	“Thinking clearly” “Clarity” “Brain fog”	Confused subscale of POMS [33] Bond- Lader VAS (Muzzy-Clear headed)	Mental clarity is a subjective phenomenon that limited tools have been developed to measure. Some versions of the POMS have a clearheaded/confused subscale that is used in nutrition research. Consumers mentioned mental clarity coinciding with numerous other terms including focus, energy, sharpness, calmness, and memory indicating that to consumers this is an overarching concept linked with numerous other cognitive and mental processes.

## Data Availability

Data are available at available at https://osf.io/xvhqj/.

## Supplementary Materials

The following supporting information can be downloaded at: https://www.mdpi.com/article/10.3390/nu16121950/s1, Figure S1: CONSORT diagram.
