# Supplementary figures and images for: Alignment of Consumers’ Expected Brain Benefits from Food and Supplements with Measurable Cognitive Performance Tests
Source: Nutrients. 2024 Jun 19;16(12):1950. doi: 10.3390/nu16121950 (PMC11206270; doi:10.3390/nu16121950)

Figure S1. CONSORT diagram.

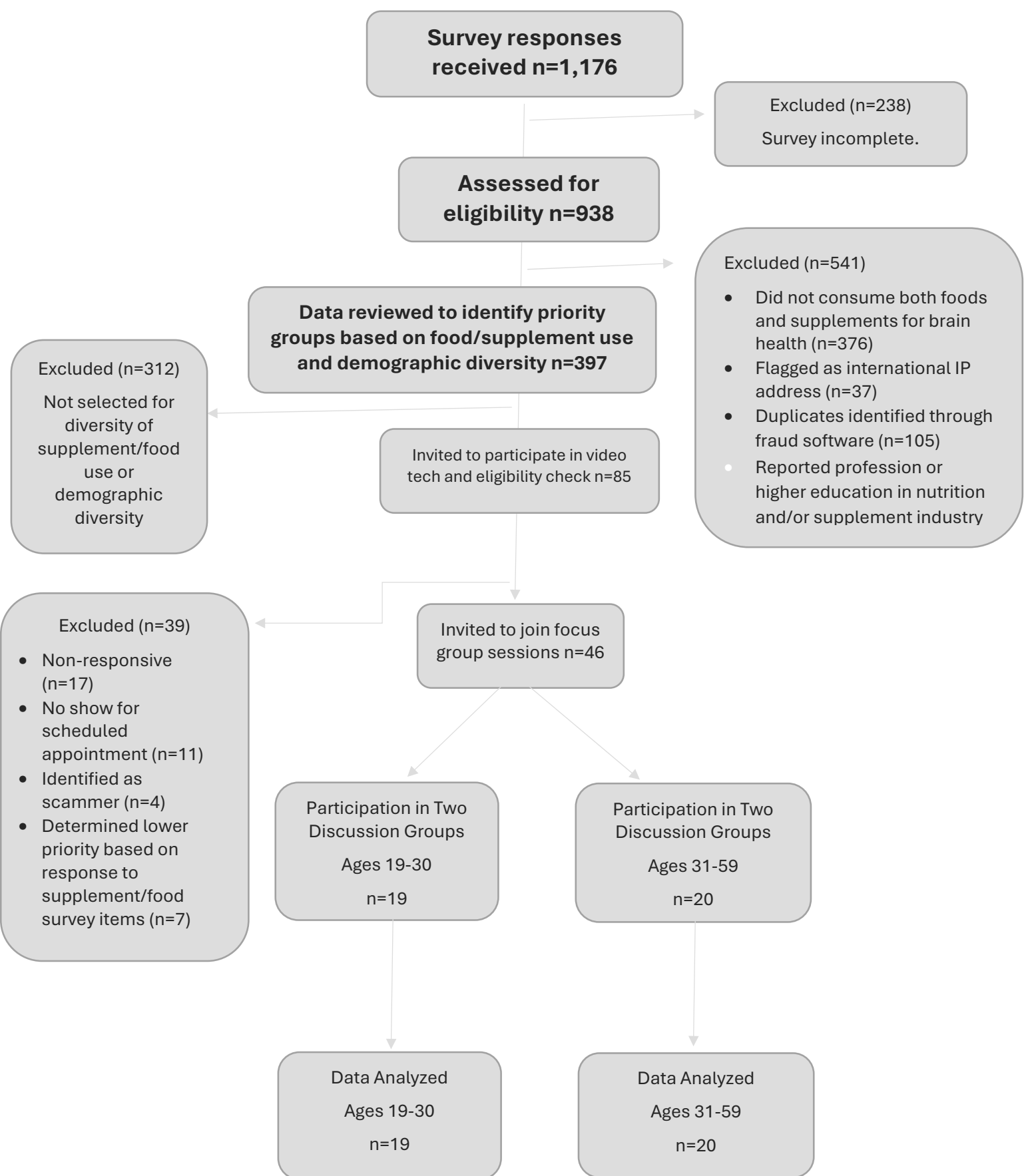

Supplement: Supplementary file 1 [file nutrients-16-01950-s001.zip › nutrients-3008584-supplementary.pdf]
